# Studies in the Medical Botany of Southern Illinois

**Published:** 1881-12

**Authors:** J. M. G. Carter


### ARTICLE IV.

### STUDIES IN THE MEDICAL BOTANY OF SOUTHERN ILLINOIS.

By J. M. G. CARTER, A.M., M.D.

### II.

The therapeutic agents mentioned in this article all belong to the botanical division called Monopetalous Exogenous Plants. The division is large, and it contains a long list of valuable remedies, many of which are found in this section of Illinois.

Several plants of the Honeysuckle Family (Caprifoliaceæ) are used in practice. Lonicera Caprifolium, containing the species L. Sempervirens, or Trumpet Honeysuckle, and L. Grata, or

Woodbine, has been prescribed, in the form of syrups, for asthma and other lung troubles. In domestic practice the juice of the plant is frequently used for the bites and stings of insects. The bruised plant may be placed on the injury as a poultice, or the juice rubbed on as a linament. The fruit of all species of Lonicera is said to be emetic and cathartic.

To this order belongs Fever-wort, Fever-root, or Wild Ipecac (Triosteum Perfoliatum, T. Augustifolium). It is also called Horse Gentian. It is a bitter tonic in small doses. In larger doses it is a diuretic; and in doses of 20 to 30 grains of the bark of the root, it is emetic and cathartic.

Sambucus Canadensis (Common Elder) has been used in domestic practice as a tonic in the form of "bitters." This member of the Caprifoliaceæ is tonic, diaphoretic, diuretic, alterative, emetic and cathartic. The juice of the berries is aperient, and has been recommended in constitutional diseases and eruptive affections as an alterative. It has been thought of especial value in rheumatic, syphilitic, and gouty diatheses. For these diseases the juice of the root or berries may be given in doses of fifteen drops four times a day. In tablespoonful doses it may be used as an emetic, or as a hydragogue cathartic in dropsy. One dose a day will probably be sufficient for either purpose. It is sometimes recommended for epilepsy. How does it act in this malady? This is a question which I cannot answer. I have not used the remedy in this disease; but I presume that whatever benefit is derived from it must be due to the valerianic acid and tannic acid contained in it. If epilepsy is due to variations in the caliber of the cerebral vessels, as has been asserted, the sedative and astringent properties of these constituents might have some slight beneficial effect.

Perhaps the most important genera of Caprifoliaceæ, considered from a medical point of view, is Viburnum. The particular plant of this genus to which I wish to call attention is V. Prunifolium (Black Haw.) This is one of the new remedies in regular practice, but the Eclectics have used it for many years. Its value as a tonic and uterine sedative has been brought before the profession by Drs. D. L. Phares, E. W. Jenks, and others. It is of especial value in threatened abortions. It may be given alone in such cases, or used in conjunction with opium. It has been recommended as antispasmodic, astringent and diuretic.

I have used this remedy in about one hundred cases of uterine or ovarian diseases, and its administration has been attended with remarkable success. I have used it in ovarian neuralgia; neuralgia of the os uteri; endometritis (especially chronic), whether general, corporeal or cervical; areolar hyperplasia; dysmenorrhœa; amenorrhœa; and hysteria; and in all forms of neuralgia and headache supposed to be due to some uterine trouble. That is, I have used it as a uterine sedative to control pains and soreness in the region of the uterus, to control leucorrhœa, pains in the back, dragging pains in front, etc.

I used it in one case of neuralgia and leucorrhœa due to lacerated cervix uteri. I prescribed the fluid extract in ʒss doses four times a day. After a month the patient stopped taking the remedy, and her improvement, which had been satisfactory, has not progressed any since; still, her present condition is better than before taking the medicine.

The value of the remedy in dysmenorrhœa may be shown by referring to the case of a young married lady (married one year) who had been suffering every month for some two years, her trouble gradually growing worse. When I was called she was in one of her severest paroxysms of pain. I gave anodynes for temporary relief, and kept her upon a mixture of V. Prunifolium and Valerian, chiefly the former, until the next monthly sickness. This time her suffering was so slight that I was not consulted. She continued taking the mixture, having no special pain or sickness next month, and has not had since—several months. A case of hysterical paroxysms, which had been increasing in frequency and severity for four years, but which yielded to the action of the Black Haw in less than three months, might be described.

Eupatorium Perfoliatum and several other varities of Eupatorium (E. Altissimum, E. Sessilifolium, E. Scrotinum), are found in S. Illinois. E. Perfoliatum is generally called Thoroughwort in the North and East, but in this section it is better known as Boneset. Boneset is one of the best-known remedies in our portion of the State. Many persons make free use of it when feeling

"bilious," dull headache, impaired appetite and general malaise. I have frequently prescribed it in such cases myself with excellent results. Sometimes I direct a tea to be made of the leaves or tops, to be taken *ad libitum.* Frequently I direct two ounces of the leaves to be boiled in a pint of water, and prescribe one or two tablespoonfuls t. d., generally before meals.

When it is desired to keep the preparation for some time, it is better to add an ounce of alcohol to each pint. It is said that the cold infusion, or the powder, is best for its tonic effects. The infusion should be warm, and the patient should be covered up in bed to obtain its diaphoretic effect. The decoction I have mentioned may be given in doses of ℥iv to ℥viij for the action of the remedy as an emetic or cathartic. It is used in intermittent, remittent and typhoid fevers.

Erigeron belongs to the same family as Eupatorium, Compositæ. The Erigeron Canadense is the most important variety or species found in Southern Illinois. I believe E. Philadelphicum and E. Annuum are found in this part of the State also, but I am not aware of any general or domestic use of them other than what is given in the books.

Golden-rod (Solidago) is another Composite of medical interest which grows abundantly in this region. The varieties found here are S. Bicolor, S. Speciosa, S. Petiolaris, S. Missouriensis, and others. This aromatic, stimulant, carminative, and diaphoretic, is not used by the common people and rarely by the profession, so far as I am aware. Ambrosia (Ragweed), two or three species of which are found, is an Eclectic remedy—a tonic in low fevers.

Helianthus (Sunflower), another of the Compositæ, I mention only because it has the reputation of protecting against malaria. It is said that malarious districts have been rendered healthy by planting H. Annuus. The pith of the same plant has been recommended for the preparation of moxa on account of the niter which it contains. To the same order belongs the Bidens Bipinnata (Spanish Needles.) This has some reputation among the people as an emmenagogue. The root and seeds of this and other species (B. Froudosa, B. Connata) are thus used, and are prescribed by the Eclectics as expectorants in laryngeal and bronchial diseases. They may be used in infusion, or better, in decoction. For its emmenagogue effect let the patient drink a teacupful of the warm tea of the root (ʒj to Oj) every two hours.

Perhaps there is no Composite of medical value which grows more abundantly than Wild Chamomile (Maruta Cotula) in this part of the State. This is the common Mayweed, known in some places as Dog Fennel. Its physiological action and its therapeutic uses are similar to Chamomile (Anthemis Nobilis), and it may be substituted for that remedy. The remedy is best administered in the form of an infusion. The bruised plant, or the infusion, have been recommended for ulcers. The application is said to cleanse the part and restore the normal color. Mayweed has been thought to be emmenagogue also. The flowers are more pleasant for internal use ; but the entire plant is active.

Tanacetum Vulgare (Common Tansy), of this order, is very common in Southern Illinois, escaped from gardens. The leaves and tops are used. The remedy is also emmenagogue. It is tonic, deobstruent and anthelmintic. Dose, ʒss to ʒj. The infusion may be used as a tea. The seeds are used as a vermifuge. The acid—tanacetic or tanisic—is prescribed for the same purpose. The remedy has been recommended in epistaxis, the odor being sufficient to check the hæmorrhage sometimes (Dr. C. P. Uhle.) Tansy has been used in intermittent fever, arthritic paroxysms, hysteria, and amenorrhea with asserted success. Tanacetic acid is equal to santonin in efficacy as an anthelmintic, and may be given in the same dose. "Tanacetic acid may be obtained by concentrating to the consistence of honey the residue after the distillation of the tops, heating with chalk and animal charcoal, and evaporating. If the residue be stirred with water, acidulated first with muriatic, and afterward with acetic acid, colored crystals of tenacetic acid are obtained, which are to be washed with distilled water."

The tansy tea is often given to girls who have "taken cold," where the menses are suppressed.

Of the Composite Lappa Officinalis (Burdock), there are three varieties in Southern Illinois (L. Major, L. Minor, L. Tomentosa.)

Burdock is a common domestic remedy. It is chiefly used as a diaphoretic; it has value, however, as an aperient and as a diuretic. It has been recommended to the profession as valuable in cutaneous diseases; as herpes, lepra, psoriasis, prurigo, acne, and the skin diseases of children. I know the case of a boy who was suffering with itch (scabies), and determined to try what virtue there was in Burdock. He gathered quite a large quantity of the root of Common Burdock (L. Major), made a strong decoction of it, put it into a tub and bathed all over. In a few minutes he was covered all over the body with great wheals, which gave him intense itching, burning and pain. However, in a few hours these symptoms passed away, and with them the itch disappeared. The treatment was severe, but it produced a radical cure.

The remedy has been recommended also in nephritic affections, and in rheumatic, gouty, venereal, scorbutic, leprous, and scrofulous troubles. It has been used as a wash for chronic ulcers. The docoction is the preparation generally used.

Monotropa Uniflora (Indian Pipe, Corpse-Plant) is said to have been used in decoction as an alterative, or a tonic, by the common people of this section of the State. I have been told that formerly it was used to a considerable extent for this purpose in "biliousness." I have made a study of the plant, and am not aware that any other physician has ever made it the subject of investigation. Indian Pipe belongs to the Heath family (Ericaceæ), order Ebenaceæ (Ebony family.) The Persimmon, of opossum fame, belongs to this family. This tree, Diospyros Virginiana (Common Persimmon, Date-plum, American Date), grows in great numbers in our section. It has been used in diarrhœa, dysentery, uterine hæmorrhage, intermittent fever, sore throat, and by the people for making beer. The fruit was used formerly for making beer to a greater extent than now. The bark has been used in intermittents, and for ulcerated throat only, so far as I know. It may be given in the form of a decoction. For diarrhœa, dysentery and hæmorrhage it may be prescribed (that is, the fruit) as syrup, tincture or infusion. The green fruit should be used, as during the maturing process the fruit undergoes some change by which it loses its astringency.

Another genus of this large family is Taraxacum. T. densleonis (Common Dandelion) is abundant in pastures and fields. This is a valuable remedy for such a region as ours, where the liver and spleen are generally involved in the various diseases with which we meet. It is especially in hepatic and splenic affections—engorgements—that it manifests a curative action. It may be used in both acute and chronic diseases of the stomach, liver and bowels.

The last plant of this order that I shall mention is Wild Lettuce. Lactuca Canadensis contains at least two varieties found in Southern Illinois (L. integrifolia, L. sanguinea.) It has been asserted by some physicians to have properties similar to garden lettuce (L. sativa), but this is denied by others. The actions which have been claimed for it are anodyne, diaphoretic and diuretic. I have not used it. The only plant of the Lobeliaceæ that I desire to mention is Indian Tobacco or Lobelia (L. Inflata.) This poisonous herb is used largely in domestic practice and by quacks, but it should not be confided entirely to their keeping. It should not be used as an emetic; but as an expectorant in asthma and bronchial diseases, cautiously administered, it is valuable. It is similar to Tobacco (Nicotiana Tabacum) in its action.

The Plantain family (Plantaginaceæ) is well represented in Southern Illinois. Plantago Major (Common Plantain) is a remedial agent of some value. It has been recommended for internal use in doses of one to four fluid ounces of the expressed juice. It is considered astringent, deobstruent, diuretic and refrigerant. The ancients used the remedy in visceral hæmorrhage and obstructions. I am only acquainted with its external use. It is freely used as a poultice in all kinds of ulcers and local inflammations, whether acute or chronic; in scrofulous tumors, and for dressing blisters. In modern times, as well as among the ancients, it has been used in consumption, dysentery, and intermittents, internally. The decoction may be used for either internal or external purposes. Externally, the leaves may be applied whole, sometimes bruised, or withered by dipping in hot water.

The Catalpa, of the Bigonia family (Bigoniaceæ), is represented by C. bigonisides. This tree, though not abundant, yet grows in some places in considerable numbers. Many of them grow near Grayville, and are valued as fencing material. Their medicinal value is not so well understood. The Catalpa (Indian Bean) has been used by some European physicians in asthma. A decoction of the seeds is recommended. The fruit, a long bean, is generally considered to be poisonous. I have not used the remedy.

Verbascum Thapsus (Common Mullein) is the only plant of the Figwort family (Scrophulariaceæ) of medical interest that grows in Southern Illinois. Its properties are demulcent, emollient, and anodyne. It has been used in chest troubles. The tea is used in domestic practice. An infusion of the flowers has been used in mild catarrhs. A decoction of the leaves is said to be useful in diarrhœa. The dose of the infusion is one or two wineglassfuls. The mullein leaves are used in this vicinity as an anodyne poultice; this is the only use of the plant that I have made. The leaves should be dipped in hot water before using for external application.

Mentha Viridis (Spearmint), M. canadensis (Wild Mint), Hedeoma Pulegioides (American Pennyroyal), Salvia Lyrata (Lyre-leaved-sage), Monarda Punctata (Horse-mint), Nepeta Cataria (Catnip), of the Mint family (Labiatæ), all grow in this region, and are used as domestic remedies, generally in the form of tea, for colds and colic. Sage tea is a favorite mouth and throat wash in ulcerated conditions of these parts. Many mothers use them freely for their daughters in dysmenorrhœa. Horehound (Marrubium Vulgare) is used in the same way, more especially in colds.

The Nightshade family (Solanaceæ) is represented in Southern Illinois by Solanum Dulcamara (Bittersweet), S. Nigrum (Common Nightshade), Physalis Pubescens (Ground Cherry), Datura Stramonium (Jamestown Weed, Thorn-apple), all of which have medical interest. Common Nightshade (S. nigrum) is a frequent domestic remedy, in the form of a poultice of the leaves and plant, to bruises, sprains, eruptions due to poisoning, etc.

Datura Stramonium is used as a poultice, domestically, over boils, carbuncles, painful tumors and local inflammations, bruises, stings of insects, etc. The latter remedy is very abundant, and the physician frequently finds it convenient to make use of this plant. The leaves are generally used for poultices. The Ground Cherry may be used in the same way, but is not so efficacious. The fruit is edible.

I do not know of any other application or use of any of the Solanaceæ not described in the books.

Frasera Carolinensis (American Columbo), of the Gentian family (Gentianaceæ) is found in this part of the State. It is also called F. walteri. This is a simple bitter, and the infusion was so used formerly by physicians and people in domestic practice. The root is the part used. It may be employed in powder or decoction. I obtained my information concerning this plant from the late Dr. C. R. Smith. I have not seen it.

A valuable remedy of the Loganiceæ (Logania family) which grows in this section is Spigelia Marilandica (Pink-root). It is used in domestic practice, and sometimes in general practice, in the form of a tea, against the round worm (Ascaris Lumbricoides.)

